# Phosphorylation of C3a Receptor at Multiple Sites Mediates Desensitization, β-Arrestin-2 Recruitment and Inhibition of NF-κB Activity in Mast Cells

**DOI:** 10.1371/journal.pone.0046369

**Published:** 2012-10-15

**Authors:** Kshitij Gupta, Hariharan Subramanian, Andreas Klos, Hydar Ali

**Affiliations:** 1 Department of Pathology, School of Dental Medicine, University of Pennsylvania, Philadelphia, Pennsylvania, United States of America; 2 Institute of Medical Microbiology and Hospital Epidemiology, Medical School Hannover, Hannover, Germany; Harvard Medical School, United States of America

## Abstract

**Background:**

Phosphorylation of G protein coupled receptors (GPCRs) by G protein coupled receptor kinases (GRKs) and the subsequent recruitment of β-arrestins are important for their desensitization. Using shRNA-mediated gene silencing strategy, we have recently shown that GRK2, GRK3 and β-arrestin-2 promote C3a receptor (C3aR) desensitization in human mast cells. We also demonstrated that β-arrestin-2 provides an inhibitory signal for NF-κB activation. C3aR possesses ten potential phosphorylation sites within its carboxyl terminus but their role on desensitization, β-arrestin recruitment and NF-κB activation has not been determined.

**Methodology/Principal Findings:**

We utilized a site directed mutagenesis approach in transfected HEK293 cells to determine the role of receptor phosphorylation on β-arrestin-2 recruitment and RBL-2H3 cells for functional studies. We found that although Ala substitution of Ser475/479, Thr480/481 residues resulted in 58±3.8% decrease in agonist-induced C3aR phosphorylation there was no change in β-arrestin-2 binding or receptor desensitization. By contrast, Ala substitution of Thr463, Ser465, Thr466 and Ser470 led to 40±1.3% decrease in agonist-induced receptor phosphorylation but this was associated with 74±2.4% decreases in β-arrestin-2 binding, significantly reduced desensitization and enhanced NF-κB activation. Combined mutation of these Ser/Thr residues along with Ser459 (mutant MT7), resulted in complete loss of receptor phosphorylation and β-arrestin-2 binding. RBL-2H3 cells expressing MT7 responded to C3a for greater Ca^2+^ mobilization, degranulation and NF-κB activation when compared to the wild-type receptor. Interestingly, co-expression of MT7 with a constitutively active mutant of β-arrestin (R169E) inhibited C3a-induced degranulation by 28±2.4% and blocked NF-κB activation by 80±2.4%.

**Conclusion/Significance:**

This study demonstrates that although C3a causes phosphorylation of its receptor at multiple sites, Ser459, Thr463, Ser465, Thr466 and Ser470 participate in C3aR desensitization, β-arrestin-2 recruitment and inhibition of NF-κB activity. Furthermore, β-arrestin-2 inhibits C3a-induced NF-κB activation via receptor desensitization-dependent and independent pathways.

## Introduction

Cross-linking of high affinity IgE receptors (FcεRI) on mast cells is known to play an important role in allergic and hypersensitive diseases [1]. Fukuoka et al [2] showed that activation of human mast cells via FcεRI results in the secretion of tryptase, which generates sufficient amount of C3a from C3 to cause mast cell degranulation. They proposed that C3a-induced mast cell activation may play an important role in mediating allergic diseases. Indeed, Shafer et al., [3] recently demonstrated that IgE-mediated passive cutaneous anaphylaxis resulted in local increase in C3a levels and that subsequent activation of C3aR in mast cells contributed to allergic skin response. Not surprisingly, we have shown that C3a causes degranulation and chemokine generation in human mast cells and in transfected RBL-2H3 cells [4], [5], [6]. However, the mechanisms involved in the regulation of C3aR signaling in mast cells remain poorly defined.

It is well established that following activation by agonists, most GPCRs are phosphorylated by a family of protein kinases, collectively known as G protein coupled receptor kinases (GRKs) [7]. Receptor phosphorylation appears to be a key mechanism by which many GPCRs are regulated. C3aR possesses ten potential phosphorylation sites within its carboxyl terminus and in transfected COS cells GRK2, GRK3, GRK5 and GRK6 promote agonist-induced receptor phosphorylation [8]. Using lentiviral shRNA-mediated silencing of GRKs in human mast cells that endogenously express C3aR, we have shown that GRK2 and GRK3, but not GRK5 or GRK6, are involved in C3aR desensitization [9]. However, the specific phosphorylation sites on C3aR that mediate receptor desensitization remain unknown.

Following agonist-induced GPCR phosphorylation, β-arrestins uncouple the receptor from G protein, leading to receptor desensitization and facilitate their clathrin-mediated internalization [10]. We have recently shown that silencing the expression of β-arrestin-2 resulted in decreased C3aR desensitization and reduced agonist-induced receptor internalization [11]. For many GPCRs, receptor internalization and β-arrestin-2 recruitment serves as a complex for the activation of ERK signaling pathways. However, we have shown that β-arrestin-2 inhibits C3a-induced ERK phosphorylation, NF-κB activation and chemokine generation [11].

The goal of the present study was to extend our previous findings with shRNA-mediated silencing of GRKs and β-arrestins in human mast cells [9], [11] and to determine the role of C3aR phosphorylation and β-arrestin-2 recruitment on desensitization, internalization and NF-κB activation in mast cells. Here, we demonstrate that although C3a causes phosphorylation of its receptor at multiple sites, Ser459, Thr463, Ser465, Thr466 and Ser470 participate in C3aR desensitization, β-arrestin-2 recruitment and inhibition of NF-κB activity. Furthermore, β-arrestin-2 inhibits C3a-induced NF-κB activation via receptor desensitization-dependent and independent pathways.

## Results

### Characterization of agonist-induced C3aR phosphorylation

**Figure 1:**
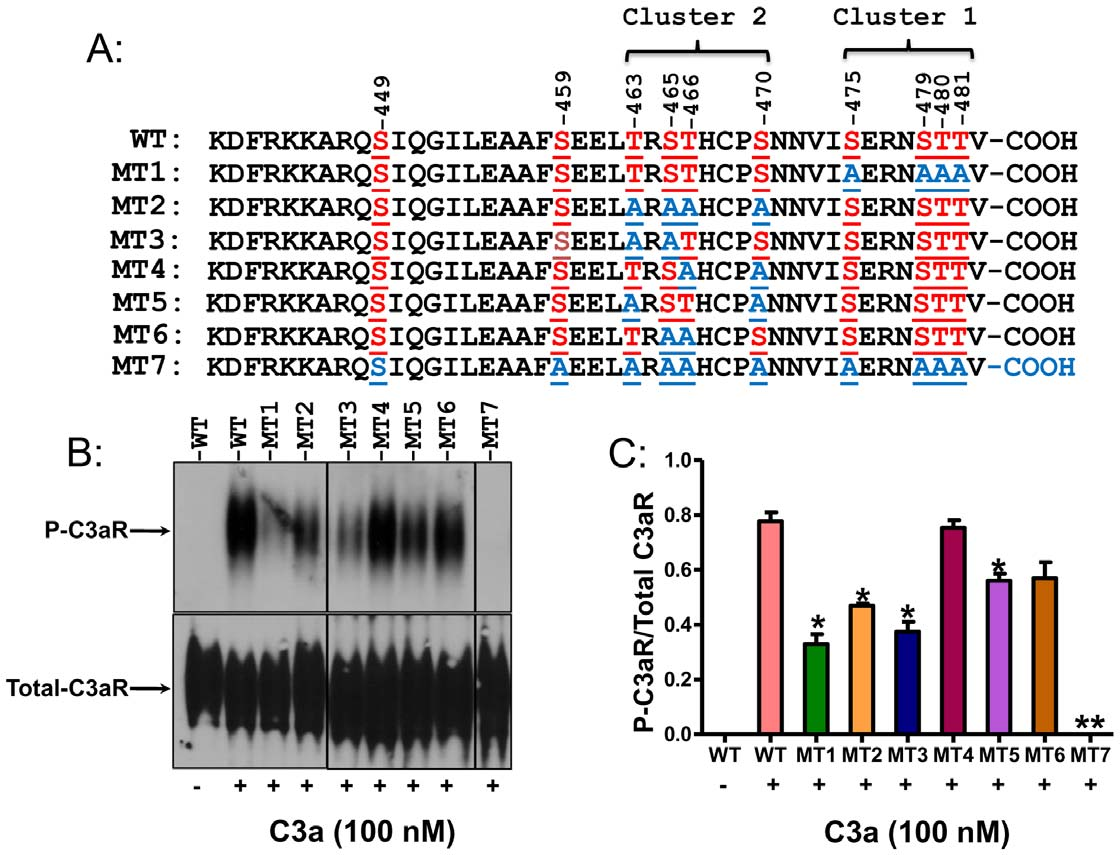
Characterization of phosphorylation sites in C3aR. (A) Schematic representation of the carboxyl-terminal domain of C3aR (WT) and mutants MT1–MT7 used for phosphorylation studies. (B) HEK293 cells transiently expressing HA-tagged C3aR or mutants were labeled with ^32^P and exposed to buffer or C3a (100 nM, 37°C for 5 min), lysed and immunoprecipitated with anti-HA-antibody, resolved by 10% SDS-PAGE and transferred onto nitrocellulose membrane. Blots were then visualized by autoradiography to determine the extent of receptor phosphorylation. Western blotting was performed with anti-C3aR antibody to determine receptor expression (bottom panel). A representative blot from three independent experiments is shown. (C) Western blotting was performed with anti-C3aR antibody to determine receptor expression. Bars represent phosphorylation of C3aR and mutants normalized to respective total receptor expression. Data represent the mean ± SEM from three independent experiments. Statistical significance was determined by two way ANOVA with Bonferroni's post-hoc test. * indicates p<0.05 and ** indicates p<0.001.

Human C3aR possesses ten potential phosphorylation sites, of which eight are present in two distinct clusters as depicted in Fig. 1A (cluster 1; Ser475/479, Thr480/481 and cluster 2; Thr463, Ser465, Thr466, Ser470). To determine their role on agonist-induced receptor phosphorylation, we initially generated two HA-tagged mutants of C3aR, MT1 and MT2, in which the Ser/Thr residues in each of the two clusters were replaced with Ala separately (Fig. 1A). We have previously used RBL-2H3 cells to transiently express C3aR for phosphorylation studies [5]. However, we were unable to express all of the mutants in this cell line at sufficiently high levels for phosphorylation studies. We therefore utilized transiently transfected HEK293 cells. These cells were labeled with ^32^P and C3a-induced receptor phosphorylation was determined. As shown in Figure 1B and C, C3a (100 nM) caused robust phosphorylation of WT-C3aR (lane 2) but this response was reduced by 58±3.8% and 40±1.3% in cells expressing MT1 and MT2, respectively. To further delineate phosphorylation sites, we focused on cluster 2 and made four additional HA-tagged mutants (Mutants MT3–MT6) by replacing two different potential phosphorylation sites at a time with Ala. We found that C3a-induced phosphorylation of MT3 (Thr463/Ser465 to Ala) and MT5 (Thr463/Ser470 to Ala) were significantly reduced when compared to the wild-type receptor. By contrast, mutations in Thr466/Ser470 (MT4) or Ser465/Thr466 (MT6) had no significant impact on agonist-induced C3aR phosphorylation. This suggests that of the Ser/Thr residues present in cluster 2, Thr463 plays an important role in agonist-induced C3aR phosphorylation.

Our next goal was to determine the role of Ser449 and Ser459, which are present outside the two main clusters of potential phosphorylation sites (Fig. 1A), on agonist-induced receptor phosphorylation. Settmacher et al, [12] showed that Ser449 participates in coupling to G proteins. Since our goal was to determine the role of receptor phosphorylation on desensitization, we did not specifically focus on Ser449. Instead, we made a mutant that incorporated Ser459 into mutants MT1 and MT2 (MT7). As shown in Fig. 1B and 1C, MT7 was completely resistant to C3a-induced receptor phosphorylation. This suggests that Ser459 co-operates with residues mutated in MT1 and MT2 to promote full receptor phosphorylation. Because mutants MT1, MT2 and MT7 contain the most important sites that are responsible for agonist-induced C3aR phosphorylation, they were used for functional studies described below.

### Role of C3aR phosphorylation on desensitization

**Figure 2:**
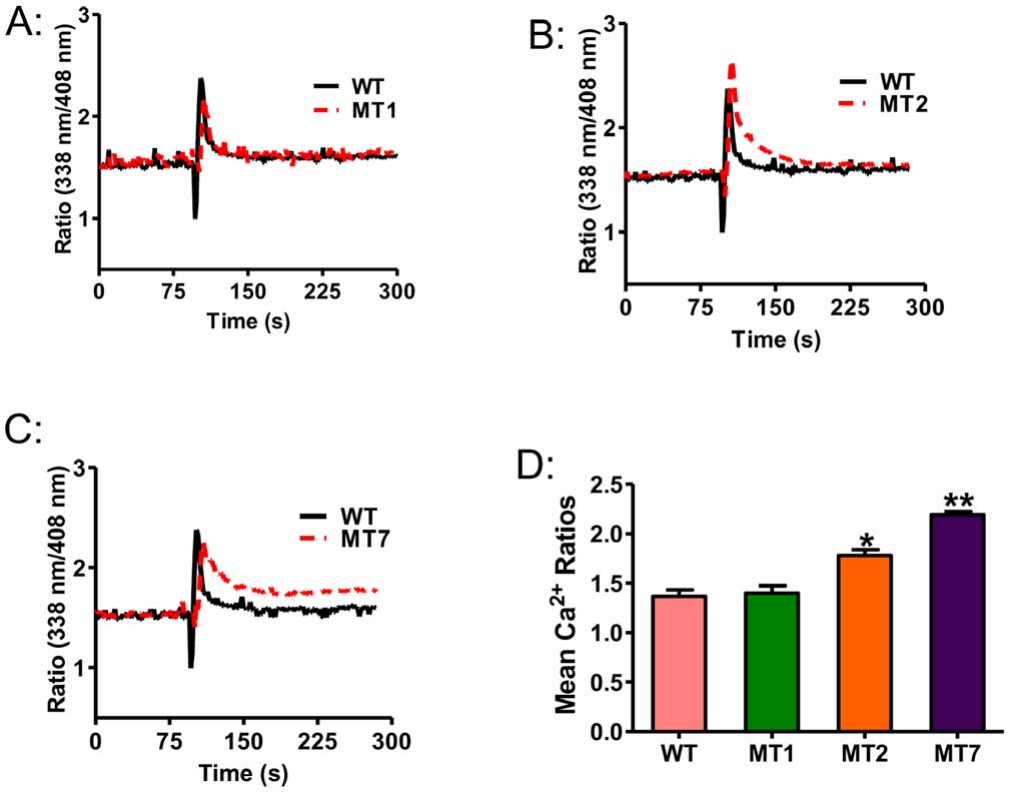
Effects of receptor phosphorylation on C3a-induced Ca^2+^ mobilization in RBL-2H3 cells. (A, B and C) RBL-2H3 cells stably expressing C3aR (Dark line) or mutants MT1, MT2 and MT7 (Red line) were loaded with Indo-1 (1 µM) and Ca^2+^ mobilization response following stimulation with C3a (100 nM) was determined. (D) Shows peak Ca^2+^ mobilization at 105–160 sec after stimulation. Data represent the mean ± SEM from three independent experiments. Statistical significance was determined by unpaired two-tailed *t* test * indicates p<0.05 ** indicates p<0.001.

Settmacher et at., [12] have utilized HEK293 cells co-expressing C3aR mutants and Gαo and assessed C3a-induced GTPγS binding in membrane preparations. They demonstrated that mutation depicted in MT2 responded to C3a for greater GTPγS binding when compared to wild-type C3aR. In addition, the ability of C3a to induce GTPγS binding was further increased membranes from cells expressing MT7. However, HEK293 cells do not respond to C3a for Ca^2+^ mobilization/degranulation and therefore cannot be used to study C3aR regulation in mast cells. We have previously performed intracellular Ca^2+^ mobilization and degranulation assays in a transfected mast cell line, RBL-2H3 cells, in order to determine the role of receptor phosphorylation on desensitization [13], [14], [15]. In this system, receptors that are resistant to desensitization, respond to ligand for more sustained Ca^2+^ mobilization and greater degranulation when compared to cells expressing wild-type receptors. To test the role of site-specific C3aR phosphorylation on desensitization, we generated stable transfectants in RBL-2H3 cells expressing HA-tagged WT-C3aR, mutants MT1, MT2 and MT7 at equivalent levels (see Method Section) and tested the effects of C3a on Ca^2+^ mobilization and degranulation. As expected, C3a caused a rapid increase in Ca^2+^ mobilization in RBL-2H3 cells expressing WT-C3aR which decayed rapidly and reached near baseline within ∼1 min (Fig. 2). Cells stably expressing mutant MT1 showed an intracellular Ca^2+^ mobilization response similar to WT-C3aR (Fig. 2A and D). By contrast, C3a caused a greater Ca^2+^ response in mutant MT2 when compared to WT-C3aR or mutant MT1 (Fig. 2B and D). Interestingly, cells expressing mutant MT7 responded to C3a for a more robust Ca^2+^ mobilization than WT-C3aR or mutant MT2 (Fig. 2; C–D).

**Figure 3:**
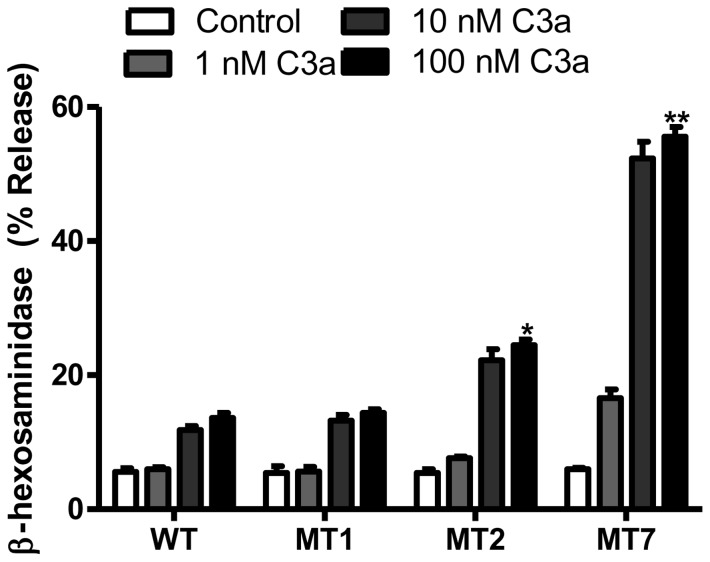
Effects of receptor phosphorylation on C3a-induced degranulation. RBL-2H3 Cells stably expressing either C3aR or mutants were exposed to buffer or different concentrations of C3a (1, 10, and 100 nM) and percent degranulation (β-hexosaminidase release) was determined. Data represent the mean ± SEM from three independent experiments. Statistical significance was determined by two way ANOVA with Bonferroni's post-hoc test. * indicates p<0.05 and ** indicates p<0.001.

**Figure 4:**
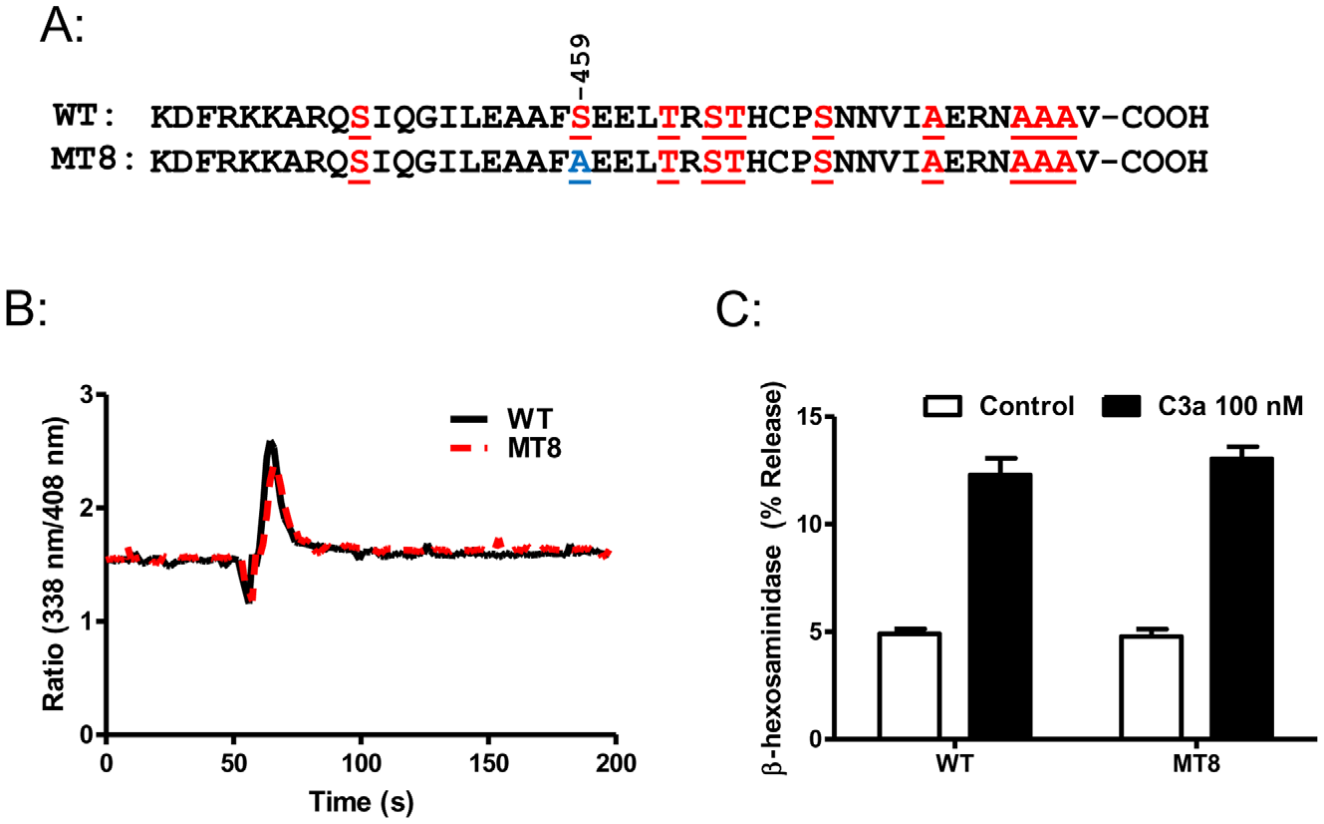
Role of Ser459 on C3a-induced Ca^2+^ mobilization and degranulation. (A) Carboxyl terminus of WT-C3aR and a point mutant of Ser459 to Ala (MT8) are shown. (B). RBL-2H3 cells stably expressing either C3aR or MT8 were loaded with Indo-1 (1 µM) and Ca^2+^ mobilization response following stimulation with C3a (100 nM) was determined. A representative traces from three independent experiments are shown. (B) Cells were exposed to buffer or C3a (100 nM) and percent degranulation (β-hexosaminidase release) was determined. Data represent the mean ± SEM from three independent experiments.

In cells expressing WT-C3aR, C3a (10 and 100 nM) caused ∼10% degranulation (Fig. 3). This response was not altered in cells expressing MT1 but was almost doubled in RBL-2H3 cells expressing MT2. However, in cells expressing MT7, C3a-induced degranulation was enhanced by >4-fold when compared to WT-C3aR (Fig. 3). Thus, studies with GTPγS binding in transfected HEK239 cells [12] and Ca^2+^ mobilization/degranulation in RBL-2H3 cells clearly demonstrate of phosphorylation sites modified in mutants MT2 and MT7 play an important role on C3aR desensitization. To determine the role of Ser459 alone on C3aR desensitization, we generated a point mutant in which this residue was replaced with Ala (MT8, Fig. 4A). RBL-2H3 cells expressing MT8 did not undergo desensitization, as cells expressing this mutant and WT-C3aR responded to C3a for similar Ca^2+^ mobilization (Fig. 4B) and degranulation (Fig. 4C). These findings suggest that phosphorylation of the receptor at Ser459 alone is not sufficient to induce desensitization but it co-operates with other sites present in cluster 1 and cluster 2 to promote a full response.

### Role of C3aR phosphorylation on β-arrestin-2 recruitment and receptor internalization

**Figure 5:**
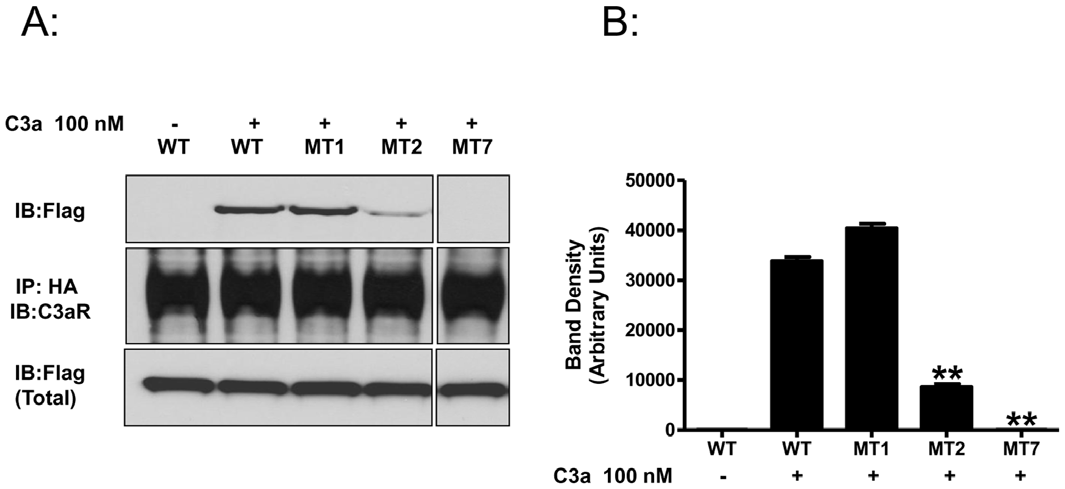
Role of C3aR phosphorylation on β-arrestin-2 recruitment. (A) HEK-293 cells transiently transfected with Flag-βarrestin-2 and HA-tagged WT-C3aR, or indicated C3aR mutants were exposed to buffer or C3a (100 nM) for 5 min. After chemical cross-linking with disuccinimidyl suberate (DSS), HA-tagged receptors were immunoprecipitated and probed with anti-Flag antibody (*Upper panel*). The membrane was then stripped and reprobed with anti-C3aR monoclonal antibody (*Middle panel*). Whole cell lysates were analyzed by Western blotting with anti-Flag antibodies to ensure equivalent expression of β-arrestin-2 in all samples (*Lower panel*). A representative blot from three independent experiments is shown. (B) Co-immunoprecipitation of β-arrestin-2 was quantified by densitometry using Image J software. Statistical significance was determined by one way ANOVA with Bonferroni's post-hoc test. * indicates p<0.05 and ** indicates p<0.001.

In addition to desensitization, agonist-induced phosphorylation of many GPCRs, results in the recruitment of β-arrestin and receptor internalization [11], [16]. We have recently shown that silencing the expression of β-arrestin-2 in human mast cells that endogenously express C3aR results in reduced receptor internalization [11]. To examine the role of C3aR phosphorylation on β-arrestin-2 recruitment, we transiently expressed HA-tagged C3aR or its mutants with Flag-β-arrestin-2 in HEK293 cells and performed co-immunoprecipitation experiments. Following C3a stimulation, WT-C3aR and MT1 associated with β-arrestin-2 to a similar extent (Fig. 5). However, interaction of β-arrestin-2 with MT2 was reduced by 74±2.4%. Mutant MT7 did not associate with β-arrestin-2 (Fig. 5).

**Figure 6:**
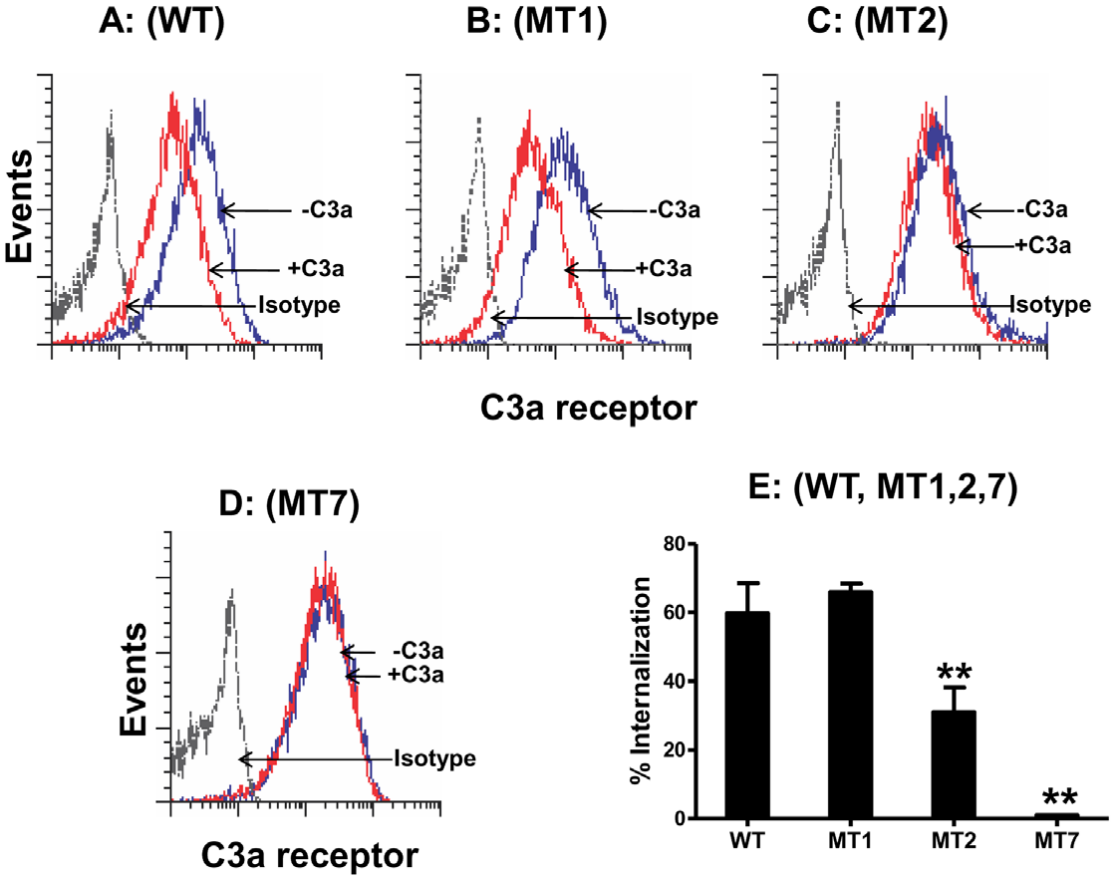
Agonist-induced receptor internalization in RBL-2H3 cells expressing C3aR and mutants. Cells stably expressing either WT-C3aR (A) or its mutants (B–D) were exposed to buffer (−C3a) or C3a (+C3a, 100 nM) for 5 min at 37°C. Cells were washed and C3aR expression was analyzed by flow cytometry. A representative histogram for each mutant from three independent experiments is shown. (E) Bar graph shows internalization of wild type and mutant C3aR expressed as the percentage loss of C3aR following exposure to C3a. Data represent the mean ± SEM from three experiments. Statistical significance was determined by two way ANOVA with Bonferroni's post-hoc test. * indicates p<0.05, ** indicates p<0.001.

Although we used HEK293 cells for receptor phosphorylation and β-arrestin-2 recruitment studies, C3aR does not undergo internalization in this system [12]. We therefore utilized RBL-2H3 cells stably expressing equivalent numbers of WT-C3aR and mutants and confirmed cell surface receptor expression by flow cytometry. C3a caused 60±8.7% and 70±2.4% internalization in cells expressing WT-C3aR and MT1, respectively (Fig. 6 A, B and E). Interestingly, internalization of C3aR was substantially reduced in RBL-2H3 cells expressing MT2 and was abolished in cells expressing MT7 receptor (Fig. 6 C, D and E).

### Role of C3aR phosphorylation and β-arrestin-2 recruitment on NF-κB activation in mast cells

**Figure 7:**
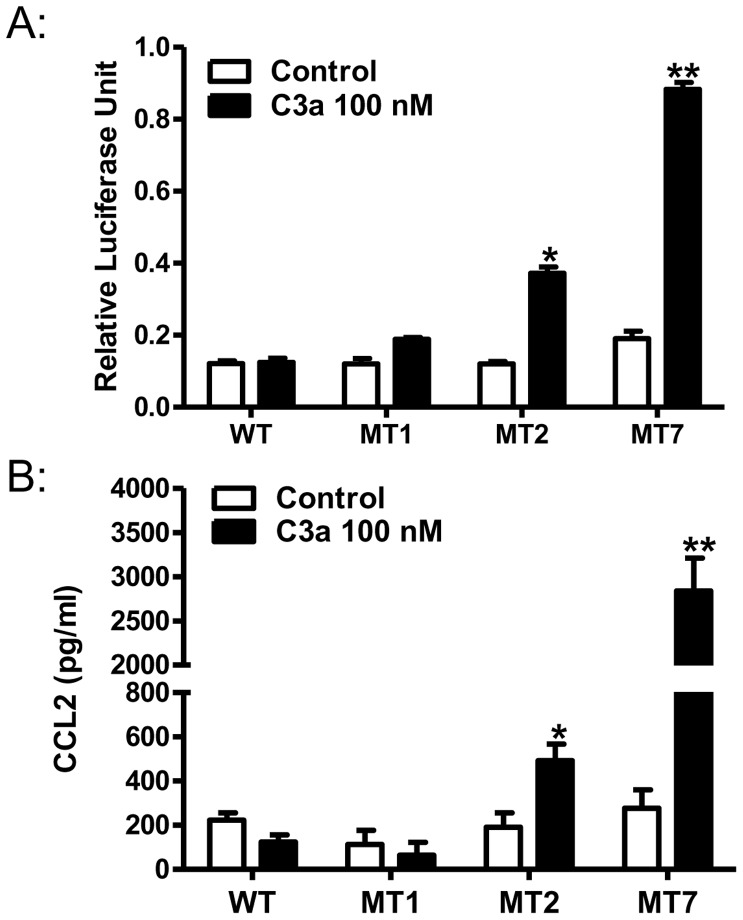
C3aR mutants MT1 and MT7 show enhanced C3a-induced NF-κB activation and CCL2 generation. RBL-2H3 cells stably expressing receptors (WT-C3aR, MT1, MT2, or MT7) were transiently transfected with NF-κB luciferase reporter gene construct. (A) Cells were stimulated with C3a (100 nM, 6 h) and NF-κB-dependent transcriptional activity was determined in cell lysates. Data presented are relative luciferase activity normalized to Renilla luciferase activity as is expressed as relative luciferase units. (B) Cells were stimulated with C3a (100 nM, 6 h) and CCL2 production was determined in the supernatant by ELISA. Data shown are mean ± SEM of three experiments performed in triplicate. Statistical significance was determined by two way ANOVA with Bonferroni's post-hoc test and unpaired two-tailed *t* test. * indicates p<0.05 ** indicates p<0.001.

C3a causes degranulation and chemokine production in a highly differentiated mast cell line, LAD2 cells [4]. However, C3a does not cause NF-κB activation and chemokine CCL2 generation in HMC-1 cells but silencing the expression of β-arrestin-2 renders the cells responsive to C3a for these responses [11]. This indicates that β-arrestin-2 inhibits C3a-induced transcription factor activation. To determine if site specific receptor phosphorylation mediates this inhibition, we transiently transfected NF-κB luciferase construct in RBL-2H3 cells stably expressing C3aR or its mutants. As shown in Fig. 7A, C3a did not induce NF-κB promoter activity in cells expressing either WT-C3aR or mutant MT1. By contrast, cells expressing MT2 showed significant enhancement of C3a-induced NF-κB promoter activity and this response was enhanced by ∼2.5 fold in cells expressing MT7 (Fig. 7A). As NF-κB plays an important role in the generation of pro-inflammatory cytokines, we next assessed the effect of receptor phosphorylation on chemokine, CCL2 generation. Consistent with NF-κB activity, C3a induced CCL2 production only in mutant MT2 and this response was greatly enhanced in cells expressing mutant MT7 (Fig. 7B).

**Figure 8:**
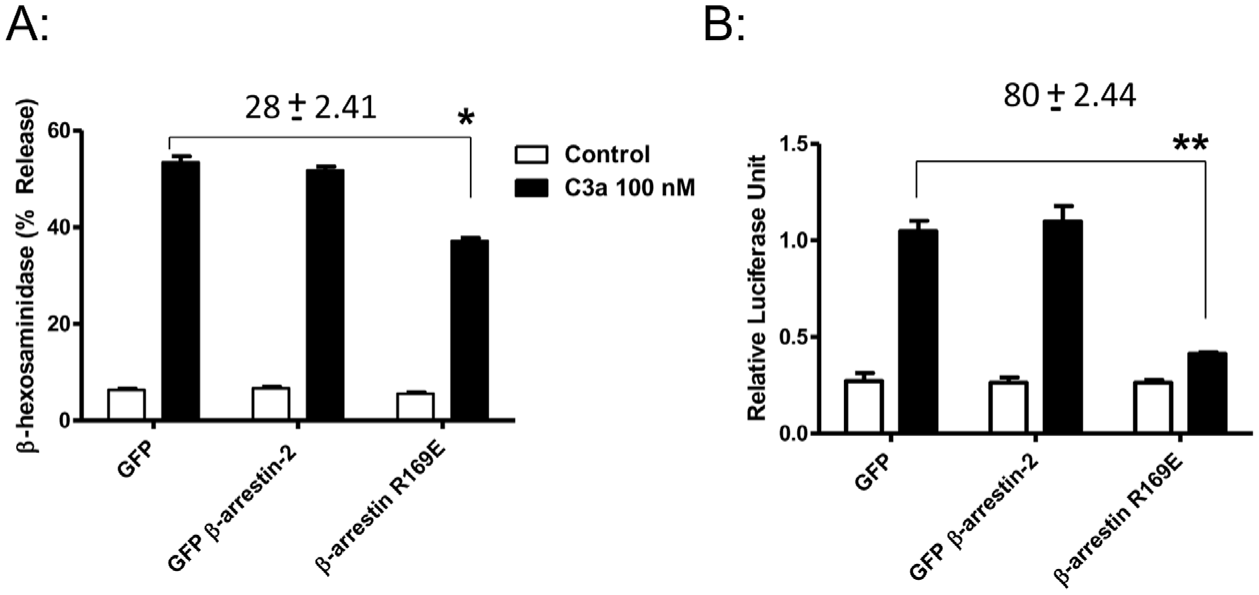
Constitutively active mutant of β-arrestin inhibits C3a-degranulation and NF-κB activation. (A) RBL-2H3 cells expressing MT7 were transiently transfected with GFP, βarrestin-2-GFP, or R169E-βarrestin-GFP. Cells were then stimulated with C3a (100 nM) for 30 min, and β-hexosaminidase release was determined. (B) Cells were also stimulated with C3a (100 nM) for 6 h, and NF-κB luciferase activity was determined. The data presented are mean ± SEM of three separate experiments performed in triplicate. Statistical significance was determined by two way ANOVA with Bonferroni's post-hoc test and unpaired two-tailed *t* test. * indicates p<0.05 ** indicates p<0.001.

Given that C3aR phosphorylation and β-arrestin recruitment promote receptor desensitization it can be speculated that enhanced NF-κB activation/chemokine generation reflects lack of receptor desensitization. To test this possibility, we took advantage of a constitutively active mutant of β-arrestin (β-arrestin-R169E) that has been shown to associate with phosphorylation-deficient mutants of various GPCRs [17], [18], [19]. RBL-2H3 cells stably expressing mutant MT7 were transiently transfected with GFP, GFP-β-arrestin-R169E or GFP-β-arrestin-2, NF-κB luciferase reporter vector and p-Renilla. NF-κB transcriptional activity was then determined after C3a stimulation using luciferase reporter assays. As shown in Fig. 8A, GFP-β-arrestin-R169E inhibited C3a-induced degranulation by 28±2.4%. Remarkably, GFP-β-arrestin-R169E inhibited C3a-induced NF-κB luciferase activity by 80±2.4% (Fig. 8B). These inhibitory effects were specific for GFP-β-arrestin-R169E, since GFP or GFP-β-arrestin-2 did not affect C3a-induced responses.

## Discussion

The anaphylatoxin C3a is generated following cross-linking of IgE-receptors on mast cells and contributes allergic responses *in vivo* by further promoting mast cell activation via C3aR [2], [3]. Accordingly, C3a induces degranulation and chemokine generation in human mast cells [4], [6]. However, the molecular mechanisms involved in the regulation of C3aR signaling in mast cells remains poorly defined. Recently, we utilized lentivirus-mediated gene silencing strategy to determine the roles of GRKs and β-arrestins on C3aR desensitization, internalization and NF-κB activation in human mast cells [9], [11]. Here, we extended these studies to identify the phosphorylation sites on C3aR that are involved in agonist-induced receptor phosphorylation, desensitization and internalization. Furthermore, we provide novel insights on the role of β-arrestin-2 on desensitization-independent signals for inhibition of NF-κB activation in mast cells.

Settmacher et al., [12] utilized a large number of phosphorylation-deficient mutants of C3aR and have shown that Ser475/479 and Thr480/481 (cluster 1) are not involved in receptor internalization but Thr463, Ser465, Thr466 and Ser470 (cluster 2) contribute to this response with Thr463 playing an important role. Given that receptor phosphorylation participates in the agonist-induced internalization of many GPCRs, our expectation was that Ala substitution of Ser475/479 and Thr480/481 (cluster 1, mutant MT1) would not affect agonist-induced receptor phosphorylation. However, we were surprised to find that these residues contributed 58±3.8% of the agonist-induced C3aR phosphorylation. Furthermore, we found that phosphorylation of C3aR at these sites did not promote β-arrestin-2 binding, receptor desensitization or internalization. The significance of C3aR phosphorylation at these sites is not known. Langkabel et al., [8] showed that in transfected COS cells although GRK5 and GRK6 enhance agonist-induced C3aR phosphorylation this has little or no effect on receptor desensitization. Furthermore, we have recently shown that silencing the expression of GRK5 and GRK6 had no effect on C3aR desensitization or internalization but rendered human mast cells more responsive to C3a for extracellular signal-regulated kinase (ERK) phosphorylation [9]. It is therefore possible that following agonist stimulation, GRK5 and GRK6 phosphorylate C3aR at one or more site within Ser475/479 and Thr480/481 to inhibit ERK signaling in the absence of receptor desensitization or internalization.

In the present study, we showed that Thr463, Ser465, Thr466 and Ser470 contribute to 40±1.3% of agonist-induced receptor phosphorylation and play a significant role on β-arrestin-2 binding, desensitization and internalization. Our phosphorylation studies with mutants MT3–MT6 indicate that Thr463 may participate in these responses. Interestingly, replacement of Ser459 to Ala alone had no effect on receptor desensitization but when combined with the eight phosphorylation sites in mutants MT1 and MT2 (Mutant M7) resulted in complete loss of phosphorylation, which was associated with substantial receptor desensitization, as demonstrated by greatly enhanced Ca^2+^ mobilization and degranulation. Unlike wild-type C3aR or MT1 and MT2, mutant MT7 did not bind β-arrestin-2 and was completely resistant to agonist-induced receptor internalization. These findings demonstrate that although C3a causes phosphorylation of its receptor at multiple sites Ser459 and Thr463 within the residues Ser459, Thr463, Ser465, Thr466 and Ser470 play particularly important role in C3aR desensitization, β-arrestin-2 recruitment and internalization.

β-arrestins have been shown to promote or inhibit NF-κB activity depending on the cell type and receptors utilized [15], [20], [21], [22], [23]. Using lentiviral-mediated gene silencing strategy in human mast cells that endogenously express C3aR, we have shown that β-arrestin-2, but not β-arrestin-1, inhibits C3a-induced NF-κB activation and chemokine generation. We have also shown that β-arrestin-2 promotes C3aR desensitization in human mast cells [11]. This suggests that receptor phosphorylation-mediated β-arrestin-2 recruitment and subsequent receptor desensitization is responsible for the inhibition of NF-κB activation. This contention is supported by the following observations. First, mutant MT1, which has no defect in β-arrestin-2 binding or receptor desensitization, responded to C3a for NF-κB activation and chemokine generation almost identical to the wild-type receptor. Second, mutant MT2 which was partially resistant to β-arrestin-2 binding and desensitization responded to C3a for enhanced NF-κB activation and chemokine generation. By contrast, MT7, which did not bind β-arrestin-2, was resistant to desensitization responded to C3a for greatly enhanced NF-κB activation and chemokine generation.

Our studies with a constitutively active mutant of β-arrestin (R169E) indicated that the ability of β-arrestin-2 to inhibit NF-κB activation involves both receptor desensitization-dependent and independent pathways. Thus, expression β-arrestin (R169E) in MT7-RBL-2H3 cells resulted in the inhibition of C3a-induced mast cell degranulation by 28±2.4%. However, under the same condition, C3a-induced NF-κB reporter activity was blocked by 80±2.4%. The mechanism by which β-arrestin inhibits the C3aR desensitization-independent transcription factor activity is unknown. Gao et al., [20] recently showed that the ability of β-arrestin-2 to inhibit cytokine production in Hela cells and THP-1 monocytes involves the formation of a signaling complex with the inhibitory IκBα. It is thus likely that internalized β-arrestin-2 and phosphorylated C3aR forms a complex with IκBα in the cytoplasm of mast cells to inhibit NF-κB activation. Because the mutant MT7 does not associate with β-arrestin2 and is resistant to internalization it probably does not form a complex with IκBα, resulting in enhanced NF-κB luciferase activity. By contrast, β-arrestin (R169E), which binds to GPCRs in the absence of receptor phosphorylation [18], [24] likely inhibits C3a-induced NF-κB luciferase activity when expressed in mutant M7 via both receptor desensitization and by forming complex with IκBα.

In summary, we have identified the phosphorylation signature within the carboxyl terminus of C3aR that mediates receptor desensitization and internalization. It is generally accepted that GPCR phosphorylation and β-arrestin recruitment to the plasma membrane promote receptor desensitization by uncoupling them from G proteins and that receptor internalization promotes downstream ERK signaling [10]. We have previously shown that β-arrestin-2 inhibits C3a-induced ERK phosphorylation possibly via its direct interaction with upstream kinases [11]. The present study extends the findings and suggests that β-arrestin-2 recruitment and C3aR internalization inhibits NF-κB activation, presumably via its interaction with IκBα. To our knowledge C3aR is the only GPCR whose phosphorylation not only desensitizes the early mast cell degranulation but also inhibits delayed ERK [11] phosphorylation and NF-κB activation.

## Materials and Methods

### Materials

All tissue culture reagents were purchased from Invitrogen (Gaithersburg, MD). Amaxa cell transfection kits and reagents were purchased from Lonza (Gaithersburg, MD). Anti- HA (HA-7) agarose beads were purchased from Sigma-Aldrich (St. Louis, MO). Anti-human C3aR antibody was obtained from Santa Cruz Biotechnology (Santa Cruz, CA). PE-labeled donkey anti-mouse IgG was purchased from eBioscience (San Diego, CA). Indo-1 AM was from Molecular Probes (Eugene, OR). SuperSignal® West Femto Maximum Sensitivity Substrate and HRP-labeled goat anti-rabbit IgG were from Thermo Scientific (Rockford, IL). Purified native C3a was purchased from Complement Technologies (Tyler, Tx). p-nitrophenyl-N-acetyl-β-D-glucosamine (PNAG) and anti-Flag monoclonal antibody were from Sigma-Aldrich (St. Louis, MO). CCL2 ELISA kit was from Peprotech (Rocky Hill, NJ).

### Cell culture

Rat basophilic leukemia, (RBL-2H3) and human embryonic kidney (HEK-293) cells (ATCC, Manassas, VA) were cultured in Dulbecco's modified Eagle's medium (DMEM) supplemented with 10% FBS, glutamine (2 mM), penicillin (100 IU/mL) and streptomycin (100 µg/mL) [25], [26].

### Construction of mutants and generation of stable cell lines

Generation of C3aR (C3aR) and C-terminal C3aR mutants in pcDNA3.1 vector were described previously [12]. HA tag was inserted into the amino termini of C3aR and mutants via subcloning into SacII and XhoI sites of pReceiver-M06 vector, which has a N-terminal 3× hemagglutinin (HA) tag (GeneCopoeia). Constructs were then verified by sequencing. RBL-2H3 cells (1×10^6^) were transfected with receptor plasmids (1 µg) using the Amaxa Nucleofector device (Amaxa kit T) according to the manufacturer's protocol. Following nucleofection, cells were cultured in the presence of G418 (1 mg/ml) and cells expressing equivalent receptors were sorted by flow cytometry using anti-C3aR specific antibody and used in subsequent studies.

### Receptor expression and phosphorylation

Transient transfections were performed on 65–70% confluent HEK-293 monolayers in 60-mm dishes with 1 µg of plasmid DNA using Lipofectamine reagent (Invitrogen). Receptor phosphorylation experiments were performed via modification of procedures described previously [15]. Briefly, HEK-293 cells expressing HA-tagged receptors were labeled with 0.15 mCi/ml [^32^P] orthophosphate for 90 min and stimulated with 100 nM C3a at 37°C for 5 min. The cells were washed with ice-cold PBS and lysed in immunoprecipitation buffer (50 mM Tris pH 8.0, 150 mM NaCl, 1.0% Nonidet P-40, 0.5% deoxycholate, 0.1% SDS, 5 mM EDTA and protease and phosphatase inhibitors). The pre-cleared cell lysates were incubated with 15 µl of anti-HA agarose beads for 2 h. Samples were washed three times with lysis buffer, resolved by 10% SDS-PAGE, transferred onto nitrocellulose membrane and ^32^P-incorporated protein bands were imaged by autoradiography. Levels of phosphorylation were normalized with their respective total levels as determined by Western blotting on the same membrane using monoclonal anti-C3aR antibody.

### Interaction of C3aR and Mutants with β-arrestin-2

HEK 293 cells transiently expressing HA-C3aR or its mutants and Flag-β-arrestin-2 were stimulated with C3a (100 nM) at 37°C for 5 min and washed with PBS. The cells were then incubated with disuccinimidyl suberate (0.1 mM) at room temperature for 30 min. The reaction was terminated by addition of ice-cold Tris-HCl (pH 7.4), to a final concentration of 10 mM and incubated for an additional 15 min. The samples were analyzed by immunoprecipitation and Western blotting. The lysates were centrifuged at 12,000 g for 15 min, and the supernatants were incubated with anti-HA agarose (Sigma) overnight at 4°C. After washing with immunoprecipitation buffer, the C3aR/β-arrestin-2 complexes adsorbed onto anti-HA agarose were eluted in sample buffer (50 mM Tris-HCl, pH 7.4, 2% SDS, 5% 2-mercaptoethanol, 10% glycerol, and 0.01% bromphenol blue), and the presence of Flag-β-arrestin-2 was detected by Western blotting analysis using anti-Flag monoclonal antibody.

### Calcium mobilization and Degranulation

Ca^2+^ mobilization was determined as described previously [27]. Briefly, RBL-2H3 cells (1.0×10^6^) were washed twice with HEPES buffer (119 mM NaCl, 5 mM KCl, 25 mM HEPES, 5.6 mM Glucose, 0.4 mM MgCl2, 1 mM CaCl_2_) containing 1 mg/ml BSA and incubated with 1 µM of Indo-1 for 30 min in dark. Cells were then washed and re-suspended in 1.5 ml of the same buffer and time course of Ca^2+^ mobilization (0–5 min) was determined using Hitachi F-2500 Fluoro spectrophotometer (San Jose, CA) with an excitation wavelength of 355 nm and an emission wavelength of 410 nm. For degranulation assay, RBL-2H3 cells (5×10^4^) were seeded into 96-well plates and incubated overnight. The following day, cells were washed twice with HEPES buffer saline and re-suspended in a total volume of 50 µl buffer containing 1 mg/ml BSA and exposed to different concentrations of C3a (1, 10 and 100 nM). For total β-hexosaminidase release, control cells were lysed in 50 µl of 0.1% Triton X-100. Aliquots (20 µl) of supernatants or cell lysates were incubated with 20 µl of 1 mM p-nitrophenyl-N-acetyl-β-D-glucosamine for 1.5 h at 37°C. The reaction was stopped by adding 250 µl of 0.1 M Na_2_CO_3_/0.1 M NaHCO_3_ buffer and absorbance was measured at 405 nm [25].

### Receptor Internalization

RBL-2H3 cells (2.5×10^5^) stably expressing WT-C3aR or mutants were stimulated with or without C3a (100 nM) at 37°C. Cells were washed twice and re-suspended in 50 µl of ice-cold FACS buffer (PBS containing 2% FBS). Anti-C3aR antibody or isotype control was added and the cells were incubated on ice for 1 h. Cells were washed twice and re-suspended in ice-cold FACS buffer. PE-labeled donkey anti-mouse was added and incubated on ice for 1 h. Cells were washed twice with ice-cold FACS buffer and fixed in 250 µl of 2% formaldehyde. Receptor internalization was quantified as the loss of cell-surface receptors, as analyzed by BD LSR II flow cytometer (BD Biosciences).

### NF-κB luciferase reporter activity and Chemokine CCL2 generation

RBL-2H3 cells (1×10^6^) stably expressing WT-C3aR or mutant C3aRs were co-transfected with NF-κB luciferase reporter gene construct (pNF-κB-LUC and p-Renilla Stratagene, Santaclara, CA) (in a 10∶1 ratio) using Amaxa Nucleofector device and kit T as per the manufacturer's protocol. After 24 h of incubation in complete medium, cells were serum starved (16 h), and then stimulated with 100 nM C3a for 6 h. Cells were washed in ice-cold PBS and finally lysed in passive lysis buffer (Promega, Madison, WI). NF-κB luciferase activity was measured using Turner biosystem 20/20 Luminometer (Promega, Madison, WI). The *firefly* luciferase activity was normalized to *Renilla* luciferase activity and is expressed as relative luciferase units (RLU). Chemokine release assay was performed as previously described [28]. For chemokine production, RBL-2H3 cells stably expressing WT-C3aR or mutant C3aRs (2×10^5^ cells) were serum starved for 4 h and stimulated with 100 nM C3a for 6 h. Supernatants were collected and stored frozen at −80°C until analysis. CCL2 chemokine levels were quantified by sandwich ELISA according to the manufacturer's protocol.

### Data analysis

The results are expressed as ± S.E.M for values obtained from at least three independent experiments. GraphPad Prism software (Graph Pad, Version 5.0 San Diego, CA) was used to analyze the data and statistical significance was determined by student's two-tailed *t*-test, one-way analysis of variance (ANOVA) with Dunnett's multiple comparison post-hoc test, and ANOVA with Bonferroni's post-hoc test.
